# Unraveling the resurgence of syphilis: a deep dive into the epidemic sweeping across the United States; a mini review

**DOI:** 10.1097/MS9.0000000000002916

**Published:** 2025-01-07

**Authors:** Zainab Fatima, Bilal Ahmad, Muhammad Abdullah, Mahammed Khan Suheb, Farheen Naaz, Aymar Akilimali

**Affiliations:** a Department of Public Health and Community Medicine, Shaikh Khalifa Bin Zayed Al Nahyan Medical and Dental College, Lahore, Pakistan; b Department of Public Health and Community Medicine, Shaikh Khalifa Bin Zayed Al Nahyan Medical and Dental College, Lahore, Pakistan; c Department of Internal Medicine, University of Wisconsin, USA; d Department of Medicine, Medical College, Deccan College of Medical Sciences, Hyderabad, India; e Department of Research, Medical Research Circle (MedReC), Goma, DR Congo

**Keywords:** congenital syphilis, prevention and control efforts, public health, sexually transmitted infection, syphilis

## Abstract

Syphilis, a resurging public health concern in the United States, has witnessed a staggering rise in cases over the past decade. This highly contagious sexually transmitted infection caused by *Treponema pallidum* presents significant challenges due to its infectious nature and potential for severe complications. Despite the successful syphilis elimination plan launched in the early 2000s, which showed a decline in syphilis cases in highly funded states, syphilis has rapidly reemerged, with incidence rates steadily climbing across many states. Transmitted through sexual contact and vertically from infected mothers to babies, syphilis progresses through distinct stages, each with varying symptoms and complications. Despite modern treatment availability such as the antibiotic benzathine penicillin G, cases often go undiagnosed until severe complications arise. Neurovascular and cardiovascular issues can result from untreated syphilis. The resurgence of syphilis is evident across demographics, with men, particularly men who have sex with men, and certain racial groups being disproportionately affected. Additionally, congenital syphilis cases have surged, leading to serious adverse outcomes for newborns. Public health initiatives must prioritize education, outreach, and preventive services tailored to high-risk populations. Partner notification, aided by technological advancements, is crucial for controlling transmission. Laboratories play a vital role in surveillance and disease monitoring. In conclusion, tackling syphilis resurgence demands a multifaceted approach addressing social, economic, and health care disparities. By implementing comprehensive public health strategies including enhanced screening and public awareness campaigns, the burden of syphilis and its associated adverse outcomes can be mitigated, safeguarding public health in the United States.

## Introduction

**Figure 1.**
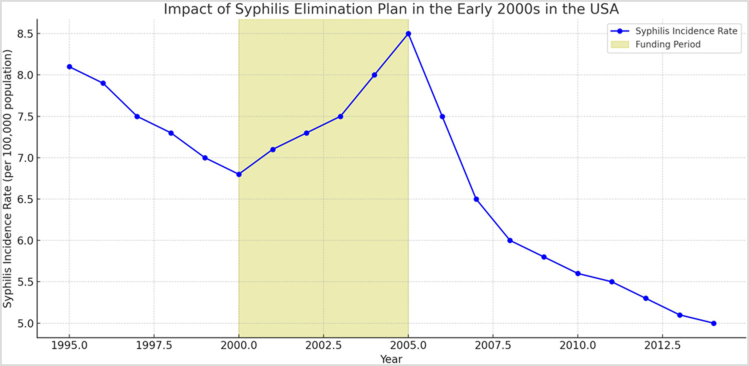
The graph above shows the impact of the syphilis elimination plan in the early 2000s in the United States. The blue line represents the syphilis incidence rate per 100 000 population over time, while the shaded yellow area highlights the funding period from 2000 to 2005.

Over the past decade, the United States has experienced an alarming rise in the cases of primary and secondary syphilis (P&S syphilis), a highly contagious sexually transmitted infection (STI) caused by the bacterium *Treponema pallidum* subspecies *pallidum*. A syphilis epidemic is occurring in the United States, with approximately 176 000 cases reported to the Centers for Disease Control and Prevention (CDC) and approximately 6 million new cases occurring across the globe. Recent statistics indicate a staggering increase in the incidence of P&S syphilis by 78.9% and congenital syphilis by 183.4%, underscoring a significant public health challenge. This resurgence exacerbates existing health disparities, especially among sexual and gender minority populations. Furthermore, its intersection with the human immunodeficiency virus (HIV)and substance use epidemics has led to a surge in congenital syphilis infections, driving up morbidity and mortality rates^[1,2]^. Notably, this resurgence has occurred despite the implementation of the syphilis elimination plan in the early 2000s, which yielded initial reductions in syphilis incidence in areas receiving funding compared to unsupported regions. Further analysis revealed that a positive correlation exists between resource allocation for syphilis elimination activities and lower syphilis rates in subsequent years. These results align with previous research linking federal sexually transmitted disease (STD) and HIV prevention funding to reduced gonorrhea rates. However, this progress was short-lived, and while evidence shows lower incidence rates following funding, the correlation does not definitively establish a causal relationship between resource allocation and long-term reduction in syphilis incidence^[3]^. Figure 1 attempts to show the impact of the syphilis elimination plan in the early 2000s in the United States by bringing attention to the syphilis incidence rate per 100 000 population over time and the funding period.

Having been nearly eradicated, syphilis has made a massive comeback, with the incidence rate increasing consistently nationwide. It is primarily transmitted through sexual contact among adults and can also be transmitted vertically from infected mothers to their infants during pregnancy or childbirth. Syphilis progresses through distinct stages over several years, each characterized by different symptoms and potential complications^[4]^.

## Clinical manifestations

Syphilis presents with a wide range of clinical manifestations, beginning with painless ulcers known as chancres, typically found on the genitals during the primary stage. As the infection progresses to secondary syphilis, patients may experience a rash, fever, and various systemic symptoms. If left untreated, the disease progresses to an early latent phase, which is characterized by positive blood tests but an absence of visible signs and symptoms, often leading to a relapse within a year. Following this, late latency occurs more than a year after infection; during this stage, the pathogen is non-infectious except in pregnancy. Tertiary syphilis, now rare due to the widespread availability of antibiotics, can lead to neurovascular, cardiovascular, and gummatous symptoms, resulting in tissue damage and cellular necrosis. Prolonged latency may also give rise to neurological complications, including tabes dorsalis and foot ischemia, underscoring the importance of early detection and treatment^[5]^.

This broad spectrum of clinical manifestations and potential complications highlights the need for a renewed focus on syphilis prevention and control in the United States. Increased public awareness and education about STIs are crucial components of effective public health strategies. This review aims to analyze the epidemiological factors contributing to the resurgence of syphilis and to recommend actionable public health measures moving forward.

Syphilis is commonly known as “the Great Imitator” due to its ability to present with clinical features resembling a variety of other conditions including herpes simplex virus, chancroid, lymphogranuloma venereum, granuloma inguinale, Reiter’s syndrome, erythema multiforme, and Behçet’s disease^[6]^. Following a period of relative decline, syphilis has experienced an alarming resurgence over the past several decades, transforming a once nearly forgotten disease into a pressing health concern that demands urgent attention and intervention. Delays in diagnosis, suboptimal patient management, and follow-up contribute to the persistence of the disease. Public health efforts at national, state, and local levels focus on syphilis prevention and control by employing a multifaceted approach consisting of disease surveillance, epidemiologic analyses, education of providers and the public, support for clinical and prevention services, outreach to recently diagnosed patients and their sex partners, and screening of high-risk individuals^[7]^.

## Epidemiology

The incidence of syphilis in the United States increased substantially from 2018 to 2022, with reported cases rising from 113 789 to 203 500 in this period. This spike reflects a concerning trend, particularly in the rates of P&S syphilis with 59 016 cases reported in 2022, corresponding to an incidence rate of 17.7 per 100 000 people. North Dakota exhibited the highest P&S syphilis rate per capita, and New Mexico reported the highest rate of congenital syphilis. Historically, syphilis incidence in the United States has displayed cyclical incidence patterns, with peaks in the early 1940s, 1980s, and a recent resurgence in the 2020s^[2,8]^.

## Demographics

Males consistently exhibit higher syphilis rates than females, especially the ones aged 30–34 years. A notable racial disparity persists, with the American Indian/Alaskan Native and Black/African American populations disproportionately affected. Geographically, the Western region reported the highest concentration of cases, followed by the South, Midwest, and Northeast. Men who have sex with men (MSM), particularly white MSM, represent a significant proportion of syphilis cases^[2]^. From 2014 to 2018, syphilis cases among MSM increased by approximately 81%. Furthermore, individuals aged 25–34 continue to account for a substantial proportion of reported cases, while young people aged 15–24, though constituting only 25% of the sexually active population, represent nearly half of all reported STIs annually^[2,9,10]^.

## Incidence of P&S syphilis

The United States has experienced a dramatic resurgence in syphilis since the early 2000s, with P&S syphilis rates increasing more than five-fold. This trend disproportionately affects vulnerable populations, particularly young people aged 15–24 years, who account for 25% of the sexually active population yet represent half of all reported STIs annually. Similarly, gays, bisexuals, and MSM (gbMSM), comprising 2–3% of the adult population, bear a significant burden, accounting for 54% of reported P&S syphilis cases. MSM experienced an 81% increase among MSM from 2014 to 2018, with a rate of 4.6 per 100 000^[2,9]^.

P&S syphilis rates have consistently risen in both men and women across all age groups and regions of the country, with a 9.3% increase from 2021 to 2022. Non-Hispanic American Indian/Alaska Native individuals experienced the largest increase, with a P&S syphilis rate of 67.0 per 100 000 in 2022. Notably, rates among women have increased since 2013, with a 19.2% national rise from 2021 to 2022, coinciding with growing cases among men who only have sex with women, reflecting an expanding heterosexual syphilis epidemic. MSM remains disproportionately affected, accounting for 45.1% of male P&S syphilis cases in 2022, with a 4.0% increase from 2021 to 2022^[2,9]^.

## Congenital syphilis incidence and overview

Congenital syphilis is a serious condition resulting from the placental transmission of *T. pallidum* from an untreated or inadequately treated pregnant female to her infant. Despite being preventable with timely screening and treatment, the rate of new infections in the United States has risen steadily since 2013^[11]^. This increase is due to multiple structural barriers that hinder both pregnant individuals and health care providers from effectively addressing the disease. It accounted for 3755 cases in 2022, including 282 congenital stillbirths and infant deaths. The national rate of congenital syphilis reached 102.5 cases per 100 000 live births, representing a 30.6% increase from 2021 and the highest reported rate since 1991. The congenital syphilis surge parallels a 17.2% rise in P&S syphilis among women aged 15–44, with 46% of counties missing the Healthy People 2030 goal. This disparity is evident in 39 states and the District of Columbia reporting increases in congenital syphilis cases^[2,8]^. Pregnant individuals, especially those from marginalized communities, face numerous barriers to prenatal care essential for congenital syphilis prevention, including poverty, substance-use stigma, citizenship status, health care inaccessibility, low sexual health literacy, and gender inequality. On the other hand, the ability of health care providers to prevent congenital syphilis is hindered by inadequate clinical guidance, decayed public health infrastructure, and patients’ multiple social challenges, such as substance use, homelessness, and mental health issues^[11,12]^. National data reveal regional variations in missed congenital syphilis prevention opportunities, with inadequate maternal treatment prevalent in the South, lack of timely prenatal care in the West, and late syphilis identification in the Northeast^[13]^.

## Comparison of syphilis incidence rates with other countries

The global landscape of syphilis trends reveals significant disparities between the United States and other countries, particularly between high-income countries and low- and middle-income countries (LMICs). Each year, around 6 million new syphilis cases occur globally among individuals aged 15–49, with a substantial burden in LMICs, where less than 25% of pregnant women are screened for syphilis.

In Africa, socioeconomic challenges and limited health care access have contributed to consistently high syphilis rates. In contrast, high-income countries, including the United States, have witnessed outbreaks primarily among MSM. European countries like Germany have reported a rise in syphilis cases, particularly among MSM, while Australia has seen similar trends, with notable outbreaks among Aboriginal and Torres Strait Islander communities. China experienced a three-fold increase in syphilis cases between 2005 and 2014, highlighting the growing epidemic among MSM and female sex workers^[14]^. Meanwhile, the United Kingdom saw a 15.2% rise in syphilis cases between 2021 and 2022, similar to the surge in the United States, where rates increased by 28.6% from 2019 to 2021^[15]^. Globally, syphilis incidence varies depending on the region. Central Sub-Saharan Africa consistently reports high rates, while South Asia has seen a decrease in age-standardized incidence rates despite increasing case numbers. This contrasts with the rising trends observed in high-income regions like North America.

The United States faces unique challenges in addressing syphilis, including disparities in health care access, especially in rural and underserved areas, which can delay timely diagnosis and treatment. Additionally, the United States struggles with higher rates of congenital syphilis, reflecting gaps in prenatal care. These factors contribute to the complex landscape of syphilis control in the United States, where, despite advances in public health efforts, the disease continues to pose a significant public health challenge^[16,17]^.

## Public health interventions pre-COVID

Before COVID-19, syphilis was largely overlooked in global health research and strategies, despite its significant impact on vulnerable populations. An 18-year analysis of infectious disease research by G20 countries showed that syphilis received the least funding relative to its burden, as measured by disability-adjusted life years. This lack of funding resulted in insufficient public health efforts, including screening and prevention, particularly among MSM and pregnant women. In the meantime, this increased the risk of HIV transmission and caused pregnancy complications, such as stillbirth. Moreover, stigma, health disparities, and weak policies further hindered effective control efforts^[18]^.

## Global impact of COVID-19 on syphilis epidemiology

The COVID-19 pandemic disrupted health care services, impacting syphilis screening, testing, and treatment, leading to a rise in cases. Public health resources were redirected to fight COVID-19, causing delays in syphilis diagnosis and care. The stigma around seeking STI services worsened, along with a continued lack of targeted research. These disruptions have contributed to the post-pandemic syphilis surge^[18]^.

## Health implications and risk factors

Despite modern treatments and preventive measures, the syphilis outbreak persists, indicating underutilization or inadequate implementation of these interventions. Social and behavioral risk factors like illicit drug use and high-risk sexual behavior highlight a lack of awareness about syphilis risks and its severe complications, including neurological issues, hearing and vision loss, and increased susceptibility to HIV. Untreated syphilis can also cause congenital syphilis, leading to stillbirth, birth defects, or infant mortality. While adult fatalities from syphilis are rare, congenital syphilis carries a high case-fatality rate. The economic burden of syphilis on the United States health care system is significant, costing about $39 million annually in direct medical expenses^[19]^. MSM are disproportionately affected by syphilis, along with other STIs like gonorrhea and HIV. Up to half of MSM in the United States use geosocial networking apps to meet sexual partners, with risky sexual behaviors linked to app usage^[20]^. Reversing the syphilis trend remains a challenge due to the stigma associated with STIs and the complexities of behavioral change. Risk factors for syphilis include age, race, number of sex partners, and drug use, underscoring the need for comprehensive public health strategies focused on prevention, early detection, and timely care^[19]^.

## Lab testing and diagnosis

Syphilis diagnosis involves both direct and indirect methods. Direct detection methods such as darkfield microscopy, polymerase chain reaction, and immunofluorescence identify *T. pallidum* in lesions. However, as these direct tests are not commercially available, indirect diagnosis through serological tests is the primary approach to syphilis detection. Serological tests are categorized into non-treponemal and treponemal tests. Non-treponemal tests, such as Rapid Plasma Reagin and Venereal Disease Research Laboratory, detect cardiolipin antibodies, while treponemal tests, including *T. pallidum* Particle Agglutination Assay and Enzyme-Linked Immunosorbent Assay, detect specific antibodies against *T. pallidum*. A two-step testing approach, combining non-treponemal and treponemal tests, is optimal for syphilis diagnosis^[12,21]^. Dual HIV/Syphilis Rapid Diagnostic Tests facilitate early diagnosis and treatment, reducing transmission risk to infants^[22]^. Laboratory reports support public health surveillance, and collaboration with local health departments and STD specialists ensures effective management and treatment^[2,8]^. However, non-treponemal tests can produce false-positive results, and treponemal tests are more sensitive but less specific, emphasizing the need for confirmatory testing^[12,21]^.

The complexity of laboratory evaluation underlines the importance of a systematic and informed approach to syphilis testing and diagnosis. Pregnancy poses additional challenges, including false-positive treponemal test results and false-negative non-treponemal tests due to high antibody levels. Monitoring non-treponemal titers regularly after treatment is essential to assess treatment response^[12]^. Despite advancements, laboratories face critical issues, including funding constraints and staffing shortages, restricting access to advanced diagnostic facilities and efficient operations. Insufficient funding limits access to cutting-edge technology, while staffing shortages lead to rising workloads and delayed test results, compromising public health responses^[15,16]^.

## Syphilis combating strategies of other nations

Countries such as Australia and Canada have implemented transformative public health interventions to control syphilis. In Australia, targeted outreach programs, particularly among MSM and Indigenous populations, have improved early detection and treatment. Canada has seen success with enhanced screening and education campaigns, particularly in high-risk populations. Both countries have utilized technology for better surveillance and contact tracing, lowering syphilis incidence. These approaches underscore the significance of tailored interventions and robust public health infrastructure^[15,16]^.

Brazil combats syphilis through effective public health campaigns, such as the “Syphilis No” project, which aims to increase national visibility and promote the public health agenda. These campaigns stimulate behavioral changes by raising awareness and encouraging individuals to seek information about testing, prevention, and treatment of syphilis. As a result of these efforts, Brazil has seen a decrease in syphilis cases; for instance, from 2018 to 2020, acquired syphilis cases dropped from 159 237 to 115 371, representing a 27.55% decrease. The growing trend of web searches for “syphilis” indicates that these campaigns have successfully reached a large portion of the target population. The United States could benefit from adopting similar strategies by investing in targeted public health communication to enhance awareness, promote behavioral changes, and reduce syphilis rates^[23]^.

Recent studies on syphilis treatment and prevention among gbMSM in urban Canada offer valuable insights into the United States context. These studies reveal significant knowledge gaps and opportunities for improved education. While 89% of Canadian participants recognized antimicrobial resistance, only 13% were aware of antibiotic-based prophylaxis, indicating a need for better education regarding prevention strategies. The belief that condom use is the only effective STI prevention method was prevalent, suggesting that awareness messaging should expand to include the benefits of prophylactic options. Importantly, a significant portion of participants (60.1%) expressed willingness to use doxycycline-based post-exposure prophylaxis (PEP) after high-risk exposure; nevertheless, interest in doxycycline-based pre-exposure prophylaxis (PrEP) was lower at 44.1%. This willingness, particularly among those who had previously used HIV PrEP, highlights the potential for integrating syphilis PrEP and PEP into existing HIV prevention efforts. US health initiatives could benefit from promoting these strategies, along with addressing misconceptions about STI risk and emphasizing the importance of regular testing and education on available prophylactic options to reduce syphilis transmission among at-risk gbMSM populations^[24]^.

## National prevention strategy for congenital syphilis

To reduce congenital syphilis, key strategies include ensuring universal access to prenatal care by addressing systemic barriers like poverty and health care gaps, reducing stigma around substance use during pregnancy, and providing better guidance to health care providers. Reinforcing public health infrastructure for tracking and treatment and integrating social support services into prenatal care are essential steps. The CDC recommends syphilis screening at the first prenatal visit, with repeat testing in high-risk areas during the third trimester and at delivery. A national prevention strategy must focus on addressing missed opportunities and ensuring timely follow-up on positive tests, providing adequate treatment to both pregnant individuals and their partners. Collaboration between public health authorities, health care organizations, and policymakers is vital to address regional differences in prevention efforts. Tailored, region-specific solutions can significantly reduce congenital syphilis cases by addressing underlying social and health care disparities. By prioritizing timely interventions, congenital syphilis in the United States can be curbed and potentially eliminated^[11,13]^.

## Strategic framework for addressing the syphilis epidemic in the United States

To effectively address the rising syphilis epidemic in the United States, especially among high-risk populations, a comprehensive strategy employing evidence-based interventions is essential. Short-term goals include implementing tailored approaches for specific populations, establishing primary and secondary prevention objectives, and harnessing technological advancements such as digital health platforms and geospatial analysis. It is also crucial to streamline the collection and analysis of surveillance data, prioritize evidence-based initiatives, and enhance interdisciplinary collaboration between health care providers and epidemiologists. Medium-term strategies focus on developing innovative primary prevention methods, assessing doxycycline-based pre-exposure and PEP regimens, and advancing research on diagnostic assays, treatment protocols, and potential vaccine candidates. Long-term goals aim to establish an effective syphilis vaccine, improve the sensitivity and specificity of diagnostic testing, and enhance STI surveillance systems. Additionally, transforming organizations through strategic workforce optimization, engaging frontline staff, and fostering inter-sectoral collaboration among health departments, academic institutions, and research entities will be critical to maximizing the collective impact of these efforts.^[25]^

## Treatment

Syphilis is readily treatable and curable with antibiotic therapy. The primary treatment regimen, endorsed by the World Health Organization (WHO), involves administering benzathine penicillin G (BPG) via intramuscular injection. For early-stage syphilis, a single BPG dose is typically efficacious, whereas later stages necessitate multiple doses, usually administered once weekly for three consecutive weeks. Alternative second-line treatments include doxycycline, ceftriaxone, and azithromycin, which may be employed in instances of penicillin allergy. Notably, BPG remains the sole WHO-recommended treatment for pregnant women with syphilis, emphasizing its critical role in preventing congenital syphilis. Prompt treatment is essential for mitigating severe health complications, particularly in cases of congenital syphilis or untreated maternal syphilis. Furthermore, intravenous or intramuscular ceftriaxone (1 g) for 10 days may serve as a viable alternative treatment option for early syphilis^[26,27]^.

## Genomics and vaccine development

A recent genomic analysis of *T. pallidum* subspecies *pallidum* revealed that this strain has significant genetic diversity, including single nucleotide variants and geographic differences in antibiotic resistance mutations. These key insights are valuable for recognizing key antigens and immune evasion mechanisms, aiding the development of vaccines and targeted immunotherapies for syphilis^[28]^.

## Prevention

Prevention of syphilis is both essential and achievable through effective public health measures. Consistent and correct use of condoms remains the most reliable method to prevent syphilis; however, the infection can still spread through contact with areas not covered by condoms, such as the genitals, anus, and mouth. Regular testing plays a crucial role, particularly for individuals at higher risk, who should undergo screening at least once a year. Pregnant women should be tested for syphilis during their first prenatal care visit, as early diagnosis and treatment with penicillin are critical in preventing congenital syphilis. Additionally, individuals diagnosed with syphilis should inform their sexual partners to prevent further transmission of the disease^[2,27]^.

## Public health efforts

Public health efforts are vital for tackling syphilis, spanning from policy advocacy to targeted interventions. By influencing policymakers, public health entities ensure financial stability for STI clinics and promote access to preventive services. Education and training for medical professionals cover prevention, diagnostics, and treatment guidelines. Collaborations with organizations serving high-risk populations enhance outreach while bridging information gaps between different program areas improves intervention effectiveness. Tailoring guidelines using local epidemiological data ensures efficient resource utilization. Partner notification remains crucial, with potential enhancements including features in dating apps mandated by the government. Network interventions target high-risk populations and locations, inducing behavioral change and providing testing opportunities. Despite budget cuts, sustained investment in public health is essential for reducing the burden of syphilis and other STIs^[19]^.

### Recommendations

Unfortunately, there is no vaccine to prevent syphilis, so the timely diagnosis and treatment of infected individuals and their sexual partners is the only key to controlling syphilis^[1,2]^. Sex education programs and the proper use of latex condoms should be taught to the general public to curb the disease. To combat syphilis effectively, medical practitioners and public health departments require increased funding for laboratory testing to facilitate early diagnosis and treatment. BPG has proven highly effective in treating syphilis^[2,8]^. However, its availability is limited in impoverished and socially disadvantaged areas, hindering access to essential care. In short, there must be sustained funding for enhanced screening, education, and treatment programs to contain syphilis spread by the policymakers and the stakeholders. Routine syphilis testing and ensuring timely treatment, particularly for high-risk groups such as MSMs, pregnant females, and sex workers, should be prioritized by health care workers. Lastly, community organizations should play a vital role in raising awareness, reducing stigma, and nurturing prevention efforts through outreach and education campaigns.

## Call to action and conclusion

To combat the resurgence of syphilis and its health implications, a coordinated, evidence-based approach is essential. First, increasing funding for syphilis screening and treatment programs is critical to improving testing availability, ensuring timely diagnoses, and providing effective treatments, especially in underserved areas where syphilis rates are high. Second, expanding health care access is paramount. Extending clinic hours, eliminating appointment requirements, and integrating syphilis testing into routine visits can enhance early detection. Telehealth services can also improve access for individuals in remote regions. Addressing social determinants of health is equally crucial for long-term success. Targeted outreach and educational campaigns for high-risk populations, such as MSM, pregnant women, and sex workers, can help combat stigma and misinformation surrounding syphilis and other STIs, fostering a supportive environment for testing and treatment. Furthermore, collaboration with community organizations and public health entities is essential in developing comprehensive prevention strategies. These strategies should include education on safe sexual practices, routine STI screenings, and condom promotion. A multifaceted approach combining funding, improved access, and attention to social factors can reduce syphilis rates and improve public health outcomes. Policymakers should increase resources, health care providers should integrate routine testing, and community organizations should lead outreach efforts. By prioritizing these measures, we can mitigate the impacts of syphilis resurgence and promote better health outcomes nationwide^[3,29]^.

To effectively address the rising rates of syphilis, several key interventions are necessary. First, implementing a hybrid resource allocation strategy that combines disease burden and population size is crucial to ensure that federal STD prevention funds are directed where they are most needed while addressing equity issues. This approach can enhance the efficiency of resource allocation and prevent over-allocation to districts that may be less effective in delivering services. High-burden districts require enhanced capacity through targeted training programs, technical assistance, and improved access to evidence-based interventions to combat STDs effectively. State and local programs should focus on improving the efficiency of their current allocations by conducting regular program evaluations to identify best practices and areas for improvement. The use of mathematical modeling can further guide the selection of target populations and interventions, as demonstrated by Australia’s “National Gay Men’s Syphilis Action Plan,” which prioritized ongoing screening and frequent testing for at-risk individuals. Incorporating recent advancements in diagnostic techniques and treatment options into public health strategies is also crucial, ensuring that health care providers remain informed about the latest innovations to enhance the effectiveness of syphilis prevention efforts. Finally, collaborative efforts among stakeholders – including policymakers, health care providers, and community organizations – are essential to developing comprehensive action plans that emphasize access to STD services for at-risk populations, ensuring alignment at all levels of the health care system in their efforts to combat syphilis effectively^[3]^.

Therefore, we issue a direct call to action to the key stakeholders to combat syphilis. Policymakers should allocate more resources toward syphilis prevention programs. Health care providers should integrate routine testing into standard care practices. Additionally, community organizations should actively engage in outreach and education initiatives to raise awareness and promote prevention. A coordinated effort among these stakeholders is vital to implement effective prevention and treatment strategies, reduce syphilis transmission, and protect vulnerable populations. Collaboration will ensure that evidence-based interventions reach those most at risk and that health care systems are equipped to respond to the growing syphilis epidemic. By working together, we can curb the spread of syphilis and improve public health outcomes. By prioritizing these public health measures, we can mitigate the impacts of syphilis resurgence and promote better health outcomes for affected populations across the nation.

Table 1 shows the CDC data of the reported cases and rates of reported cases by state/territory and regions of the United States in alphabetical order from 2018 to 2022^[2]^.

**Table 1:** **Total syphilis – reported cases and rates of reported cases by state/territory and region in alphabetical order, United States, 2018–2022(according to the Centers for Disease Control and Prevention**)**a**

State/Territory	Cases					Rates per 100 000 population				
	2018	2019	2020	2021	2022	2018	2019	2020	2021	2022
Alabama	1285	1634	1518	2173	3088	26.3	33.3	30.2	43.1	60.9
Alaska	113	242	361	447	424	15.3	33.1	49.2	61.0	57.8
Arizona	3251	4025	4461	6331	7496	45.3	55.3	62.4	87.0	101.9
Arkansas	964	1106	1243	2403	2817	32.0	36.6	41.3	79.4	92.5
California	25 253	28 811	26 414	31 280	33 346	63.8	72.9	66.8	79.7	85.4
Colorado	1085	1434	1785	2303	3100	19.0	24.9	30.9	39.6	53.1
Connecticut	264	482	536	889	760	7.4	13.5	14.9	24.7	21.0
Delaware	129	216	222	295	435	13.3	22.2	22.4	29.4	42.7
District of Columbia	764	1085	988	870	1275	108.8	153.7	143.3	129.8	189.8
Florida	10 701	12 121	12 416	16 439	18 838	50.2	56.4	57.6	75.5	84.7
Georgia	4937	5685	5595	6711	7361	46.9	53.5	52.2	62.1	67.5
Hawaii	210	252	397	643	606	14.8	17.8	27.3	44.6	42.1
Idaho	134	149	184	270	351	7.6	8.3	10.0	14.2	18.1
Illinois	4472	4511	4568	5124	5734	35.1	35.6	35.7	40.4	45.6
Indiana	985	993	1349	1980	2129	14.7	14.7	19.9	29.1	31.2
Iowa	286	359	501	763	886	9.1	11.4	15.7	23.9	27.7
Kansas	495	565	539	803	958	17.0	19.4	18.3	27.4	32.6
Kentucky	881	1096	1143	1559	2032	19.7	24.5	25.4	34.6	45.0
Louisiana	2744	2744	2497	3480	4453	58.9	59.0	53.6	75.3	97.0
Maine	147	136	81	135	154	11.0	10.1	5.9	9.8	11.1
Maryland	2536	2779	2683	NR	2798	42.0	46.0	43.4	—	45.4
Massachusetts	1305	1844	1658	2051	2444	18.9	26.8	23.6	29.4	35.0
Michigan	1692	1905	2059	2671	2824	16.9	19.1	20.4	26.6	28.1
Minnesota	918	1127	1098	1465	1839	16.4	20.0	19.2	25.7	32.2
Mississippi	1454	2006	2131	2605	3260	48.7	67.4	72.0	88.3	110.9
Missouri	1914	2188	2332	3780	4176	31.2	35.7	37.9	61.3	67.6
Montana	104	140	101	225	629	9.8	13.1	9.3	20.4	56.0
Nebraska	219	291	269	476	653	11.4	15.0	13.7	24.2	33.2
Nevada	2000	2356	2218	3065	3610	65.9	76.5	71.4	97.5	113.6
New Hampshire	137	135	120	145	175	10.1	9.9	8.7	10.4	12.5
New Jersey	1777	2085	2386	3389	3615	19.9	23.5	25.7	36.6	39.0
New Mexico	812	1294	1496	2069	2469	38.8	61.7	70.6	97.8	116.8
New York	10 183	10 500	10 613	13 106	13 685	52.1	54.0	52.5	66.1	69.5
North Carolina	2989	3369	3714	5030	6587	28.8	32.1	35.6	47.7	61.6
North Dakota	84	97	91	106	128	11.1	12.7	11.7	13.7	16.4
Ohio	1909	2005	2457	3958	5300	16.3	17.2	20.8	33.6	45.1
Oklahoma	1137	1749	1888	3003	3501	28.8	44.2	47.7	75.3	87.1
Oregon	1032	1245	1320	2010	2393	24.6	29.5	31.2	47.3	56.4
Pennsylvania	2414	2764	2898	3816	4486	18.8	21.6	22.3	29.4	34.6
Rhode Island	284	423	315	567	516	26.9	39.9	28.7	51.8	47.2
South Carolina	1152	1306	1681	2079	2473	22.7	25.4	32.8	40.1	46.8
South Dakota	74	86	128	924	1947	8.4	9.7	14.4	103.2	214.0
Tennessee	1726	2226	2463	3181	3874	25.5	32.6	35.6	45.6	54.9
Texas	12 974	12 659	15 362	21 480	26 985	45.2	43.7	52.7	72.7	89.9
Utah	423	431	351	531	673	13.4	13.4	10.7	15.9	19.9
Vermont	29	24	23	16	5	4.6	3.8	3.6	2.5	0.8
Virginia	2039	2071	1953	2205	2962	23.9	24.3	22.6	25.5	34.1
Washington	1911	2186	2079	3366	4410	25.4	28.7	27.0	43.5	56.6
West Virginia	185	277	407	536	610	10.2	15.5	22.7	30.1	34.4
Wisconsin	509	569	835	1615	1919	8.8	9.8	14.2	27.4	32.6
Wyoming	42	42	32	43	66	7.3	7.3	5.5	7.4	11.4
United States, Total	115 064	129 825	133 959	176 733	207 255	35.2	39.6	40.4	53.2	62.2
Northeast	16 540	18 393	18 630	24 114	25 840	29.5	32.9	32.3	42.2	45.3
Midwest	13 557	14 696	16 226	23 665	28 493	19.8	21.5	23.5	34.4	41.4
South	48 597	54 129	57 904	76 371	93 349	39.0	43.1	45.9	60.0	72.5
West	36 370	42 607	41 199	52 583	59 573	46.6	54.4	52.4	66.8	75.7
American Samoa	0	0	0	0	0	0.0	0.0	0.0	0.0	0.0
Commonwealth of the Northern Mariana Islands	2	2	2	2	4	3.8	3.8	3.9	3.9	7.8
Guam	30	31	21	22	19	17.9	18.4	12.5	13.0	11.2
Puerto Rico	1089	949	829	1132	1424	33.1	29.7	25.2	34.7	44.2
US Virgin Islands	NR	45	40	42	33	—	42.2	37.6	39.7	31.3
Territories Total	1121	1027	892	1198	1480	31.4	28.8	24.4	32.9	41.2
Total	116 185	130 852	134 851	177 931	208 735	35.1	39.4	40.2	53.0	62.0

^a^ Sexually Transmitted Infections Surveillance, 2022 CDC I cdc.gov [Internet]. Available from: https://www.cdc.gov/std/statistics/2022/default.htm

## Data Availability

Not applicable.
